# Multicenter Cohort Study, With a Nested Randomized Comparison, to Examine the Cardiovascular Impact of Preterm Preeclampsia

**DOI:** 10.1161/HYPERTENSIONAHA.121.17171

**Published:** 2021-08-30

**Authors:** Fergus P. McCarthy, Jamie M. O’Driscoll, Paul T. Seed, Anna Placzek, Carolyn Gill, Jenie Sparkes, Lucilla Poston, Mike Marber, Andrew H. Shennan, Basky Thilaganathan, Paul Leeson, Lucy C. Chappell

**Affiliations:** From the Department of Women and Children’s Health (F.P.M., P.T.S., C.G., J.S., L.P., A.H.S., L.C.C.), King’s College London, London, United Kingdom; Cardiovascular Division (M.M.), King’s College London, London, United Kingdom; Department of Obstetrics and Gynaecology, The INFANT Research Centre, University College Cork, Cork University Maternity Hospital, Ireland (F.P.M.); School of Psychology and Life Science, Canterbury Christ Church University, Kent, United Kingdom (J.M.O.); Department of Cardiology, St George’s University Hospitals National Health Service Foundation Trust, London, United Kingdom (J.M.O.); National Perinatal Epidemiology Unit (NPEU), Nuffield Department of Population Health (A.P.), University of Oxford; Oxford Cardiovascular Clinical Research Facility, Radcliffe Department of Medicine (P.L.), University of Oxford; Fetal Medicine Unit, St George’s University Hospitals National Health Service Foundation Trust and Molecular & Clinical Sciences Research Institute, St George’s University of London, United Kingdom (B.T.).

**Keywords:** cardiovascular diseases, dyslipidemia, heart disease, myocardial ischemia, pregnancy

## Abstract

This study evaluated whether planned early delivery would ameliorate cardiovascular dysfunction 6 months postpartum, compared with usual care with expectant management, in women with late preterm preeclampsia. We conducted a mechanistic observational study in women with preterm preeclampsia between 34^+0^ and 36^+6^ weeks’ gestation, nested within a randomized controlled trial of planned early delivery versus expectant management (usual care), in 28 maternity hospitals in England and Wales. Women were followed up 6 months postpartum with cardiovascular assessments. The primary outcome was a composite of systolic and diastolic dysfunction (by 2009 and 2016 definitions of diastolic dysfunction). Between April 27, 2016, and November 30, 2018, 623 women were found to be eligible, of whom 420 (67%) were recruited. One hundred thirty-three women were randomized to planned delivery, 137 women were randomized to expectant management within the trial, while 150 women received expectant management outside of the trial. 321 (76.4%) completed their 6 month echocardiography assessment. 10% (31/321) had a left ventricular ejection fraction <55% while 71% (229/321) remained hypertensive. There were no differences in the primary outcome between the 2 randomized groups (planned delivery versus expectant management) using either the 2009 (risk ratio, 1.06 [95% CI, 0.80–1.40]) or 2016 definitions (risk ratio, 0.78 [0.33–1.86]). In conclusion, we demonstrated that late preterm preeclampsia results in persistence of hypertension in the majority and systolic LV dysfunction in 10%, of women 6 months postpartum. Planned early delivery does not affect these outcomes. Preeclampsia is not a self-limiting disease of pregnancy alone.

## Abstract 2

Supplemental Digital Content is available in the text.

**See Editorial, pp 1395–1397**

Preeclampsia affects 3% to 5% of pregnancies complicating ≈35 000 pregnancies in the United Kingdom every year. Cardiovascular disease is the leading cause of mortality in women in the United Kingdom.^1^ Hypertensive disorders of pregnancy, in particular preterm preeclampsia, have been shown to be associated with an increased risk of developing a wide range of cardiovascular diseases, with increases in incidences observed as soon as one year postpartum.^2^ The absolute risk that a woman with preeclampsia would develop a cardiovascular event including hypertension, ischemic heart disease, stroke, or venous thromboembolism aged 50 to 59 is estimated to be 17.8% compared with 8.3% in those without preeclampsia.^3^ The American Heart Association now recognizes preeclampsia as an independent risk factor for future cardiovascular disease.^4^ The economic burden of cardiovascular disease is substantial; the British Heart Foundation estimated that in 2006 cardiovascular disease cost the National Health Service in the UK £14.3 billion and the UK economy £30.6 billion,^5^ and the costs for EU health care systems related to cardiovascular disease is estimated at €110 billion, ≈10% of the total health care expenditure across the EU. With an increasingly ageing population, costs are set to rise further.

To date, only case-control studies and small, single-center cohort studies exist to provide evidence of association between preeclampsia and persistent cardiovascular dysfunction. While some women have preexisting risk factors for preeclampsia that also predispose to cardiovascular disease, for whom preeclampsia may accelerate progression through adding a further stress, in others, preeclampsia occurs with no preexisting factors and may be the first hit in the pathway. There are several possible explanations for the additional cardiovascular insult from preeclampsia. These include subclinical myocardial injury, an increased susceptibility to undiagnosed chronic hypertension and subclinical atherosclerosis and dyslipidemia.^6,7^ This proposed research seeks to elucidate the role and extent of the myocardial stress in determining subsequent cardiac dysfunction.

We conducted a multicenter study of women with late preterm preeclampsia to evaluate the development of cardiovascular dysfunction at 6 months postpartum and to examine the cardiovascular effects of planned early delivery (compared with expectant management).

## Methods

### Study Design and Participants

A woman was eligible for the study if she was between 34^+0^ weeks and 36^+6^ weeks of gestation, had a diagnosis of preeclampsia or superimposed preeclampsia (as defined by the International Society for the Study of Hypertension in Pregnancy),^8^ with a singleton or dichorionic diamniotic twin pregnancy and at least one viable fetus, was aged 18 years or older, and was able to give written informed consent. The only exclusion criterion to study participation was if a decision had already been made to deliver within the next 48 hours. There were no substantial changes to the published study design, methods, or outcomes after the start of the trial. The study was approved by the South Central—Hampshire B Research Ethics Committee (no. 13/SC/0645) and was nested within a larger trial of planned delivery versus expectant management in women with late preterm preeclampsia (PHOENIX [Preeclampsia in Hospital: Early Induction or Expectant Management] trial; ISRCTN01879376). The data that support the findings of this study are available from the corresponding author upon reasonable request.

### Procedures

Site research teams approached women to confirm eligibility and provided verbal and written information. A trained research midwife or clinician obtained written informed consent. A research team member entered baseline data on a web-based database. All other aspects of pregnancy management were expected to be in accordance with the UK national guidelines at the discretion of the responsible clinician.^9^ Usual care in the UK was expectant management until clinical concerns led to delivery or until 37 weeks’ gestation was reached. For those women participating in the trial, they were randomly assigned to planned delivery or expectant care in a 1:1 ratio as previously described.^10^ The allocation was not masked from women, clinicians, or data collectors due to the nature of the intervention, but the study echocardiographer (J. O’Driscoll) remained masked to randomization group.

Outcomes were recorded on the web-based trial database through case-note review by trained researchers after maternal primary hospital discharge. Women were invited to return to their local hospital at least 6 months following delivery for echocardiography assessment, performed within an 8-week window of the 6-month timepoint. At this assessment, a brief medical history was recorded, blood pressure (BP) assessed, venepuncture, and echocardiography undertaken. Echocardiography was performed locally according to a standard operating procedure circulated by the research team. Echocardiography discs with anonymized participant information were then sent to the lead echocardiographer (J. O’Driscoll) who analyzed each echocardiogram without knowledge of trial allocation, entering results onto the web-based trial database. Every tenth echo was second read, again masked to trial allocation, by an echocardiographer at the University of Oxford and findings compared by the trial lead cardiologist (P. Leeson) to ensure consistency. When echocardiography assessment demonstrated potentially concerning features that may impact on clinical care, the findings were escalated and reviewed by the lead cardiologist (P. Leeson) and communicated back to the lead clinician at the recruiting site with a recommendation for clinical follow-up.

### Outcomes

The primary outcome was a composite of diastolic and systolic function at 6 months’ postpartum classified according to the joint recommendation by the American Society of Echocardiography and the European Association of Cardiovascular Imaging as assessed by transthoracic echocardiography with tissue Doppler studies classified originally in 2009^11^ (at the time of study inception) and reclassified before study completion in 2016.^12^ The primary outcome was chosen to reflect the subclinical myocardial injury in preeclampsia. Additional secondary outcomes included the cardiovascular components of a maternal morbidity composite outcome used in the main trial (severe hypertension post randomization; systolic BP ≥160 mm Hg on at least one occasion), positive inotropic support, infusion of a third parenteral antihypertensive drug, myocardial ischemia or infarction, SpO2 <90%, ≥50% FiO2 for >1 hour, intubation (other than for caesarean section), and pulmonary edema. The composite was chosen as an internationally accepted validated method for describing adverse maternal outcome from preeclampsia.^13^

### Echocardiographic Assessment

All participants were studied by standard 2-dimensional and Doppler transthoracic echocardiography at rest. Women were studied in the left lateral decubitus position and data acquired at end expiration from standard parasternal/apical views using a GE Vivid or Philips scanner.^11,14^ For each acquisition, 3 cardiac cycles of noncompressed data were stored in cine-loop format and analyzed masked to group allocation by one investigator (J. O’Driscoll), with second read as described above. Cardiac indices were normalized for body surface area, height, and end-diastolic left ventricle (LV) long or short axis lengths, as appropriate.^15–17^ Tissue Doppler imaging, strain, and strain rate indices are given as absolute values.

### Heart Remodeling

Chamber quantification and left ventricular geometric pattern were estimated using M-mode as previously described.^14^ Proximal septal bulging was assessed in the parasternal long-axis and apical 4-chamber views.^18^

### Systolic and Diastolic Dysfunction

Global LV diastolic function, estimated left heart filling pressures, and geometry was assessed and graded using standard diagnostic algorithms with the recommended adjustments reflecting the concomitant systolic function and age.^19^ LV volumes and ejection fraction were derived from Simpson’s modified biplane method from apical 4-chamber and 2-chamber views, and LV systolic dysfunction was defined as ejection fraction <55%.^14^ Hemodynamic and systolic cardiac indices were calculated as described before.^20^ Longitudinal and radial systolic function were assessed both globally and regionally using color and pulsed tissue Doppler velocity indices and strain rate indices using speckle tracking as previously described.^21–25^ Regional peak systolic strain rate index was considered abnormal if it was 2 SDs below the expected mean for age.^26^ This abnormality was defined as segmental myocardial impaired contractility. Regional diastolic dysfunction was defined as early to late strain rate ratio <1. This abnormality was defined as segmental impaired myocardial relaxation. Maternal BP was measured following the recommendations of the International Society for the Study of Hypertension in Pregnancy and National High Blood Pressure Education Programme Working Group on High Blood Pressure in Pregnancy.^27^

LV global systo-diastolic dysfunction was defined as LV global diastolic dysfunction in the presence of reduced ejection fraction (<55%). Right heart function and remodeling was assessed using integrating conventional echocardiographic indices and tissue Doppler velocity and deformation indices following published guidelines. The severity of left and right ventricle hypertrophy and dysfunction was graded according to the European Association and American Society of Echocardiography guidelines^11,14^ with the following adjustments described by Melchiorre et al^27^: age; increased circulating volume in pregnancy and the acute nature of preeclampsia on a previously normal cardiovascular system. For our primary outcome, diastolic dysfunction was classified as; normal; impaired myocardial relaxation with normal LV end diastolic pressure (GRADE I); pseudonormal filling pattern (GRADE II); and restrictive pattern (GRADE III) as previously described.^23^ Findings were also reported according to American Society of Echocardiography/European Association of Cardiovascular Imaging 2016 guidelines, published after study conception and start of recruitment.^28^

Secondary outcomes included systolic BP and diastolic BP at 6 months postpartum, together with the cardiovascular components of a composite maternal morbidity outcome adapted from the fullPIERS prediction of adverse events in preeclampsia study.^13,29^

### Myocardial Necrosis Assessment

Participants were also consented to at least 2 blood sampling time points, most commonly performed at initial recruitment and 6 month postpartum assessment. These samples were analyzed for markers of myocardial necrosis/ischemia: high sensitivity cTnI (cardiac troponins), with a sex specific level of >16 ng/L considered elevated.^30^ At 6 months postpartum, cMyC (cardiac myosin binding protein C; using Singulex’s Single Molecule Counting Technology SMC a quantitative fluorescent sandwich immunoassay technique) and NT-proBNP (N-terminal pro-B-type natriuretic peptide; Alere NT-proBNP for Alinity) were also assessed.

### Statistical Analysis

Based on previous studies of postpartum echocardiography, assuming an anticipated incidence of 70% of women with preterm preeclampsia having evidence of systolic and diastolic dysfunction at 6 months’ postpartum,^23,31,32^ a sample size of 322 women were needed to detect a 25% relative risk reduction (from 70% to 52.5%; deemed clinically important) in the primary outcome in the planned delivery group compared with those managed expectantly with a 2 sided 5% significance level and 90% power. With 20% loss of women in follow-up the overall target for recruitment was 404 women. The primary analysis for all maternal outcomes was by intention to treat with participants analyzed in the groups to which they were assigned regardless of protocol noncompliances.

Risk ratios (RRs) were estimated for binary outcomes with associated 95% CIs. Simple and multiple regression analysis were used to assess the influence of early pregnancy factors including BP, demographic variables (maternal age, body mass index), pregnancy characteristics (parity, gestation at delivery, gestation at onset, and severity of preeclampsia), on indices of cardiac function and remodeling as detailed above under proposed outcome measures. All of the conventional echocardiographic indices were adjusted for body surface area^14^ and all of the tissue Doppler velocity and deformation indices to the end-diastolic left or right ventricle long axis length.^15^ Prespecified subgroup analyses were performed for co-primary outcomes in view of changes to definitions of systolic and diastolic dysfunction over the study period. Data analyses and power calculations were performed using STATA/SE version 15.1.

## Results

### Participants

Between April 27, 2016, and November 30, 2018, 623 women were found to be eligible, of whom 420 (67%) were recruited, across 28 maternity units in England and Wales. Of these 420, 133 women were randomly allocated to planned delivery, 137 women were randomly allocated to expectant management, and 150 received usual care and were managed expectantly as indicated by national guidelines (Figure). In total, 99 (23.6%) of women did not attend their 6-month echocardiography appointment; 321 women (76.4%) completed their 6-month follow-up and had cardiac echocardiography. For the intention-to-treat analysis, data from 100 women in the planned delivery group and 107 women in the expectant management group were included. Follow-up to 6 months postpartum assessments continued until 20 June 2019. Baseline characteristics appeared similar between the 2 groups, with groups well balanced on minimization factors (Table 1). Women who did not attend their 6-month follow-up (n=99) were similar in baseline characteristics compared with women who completed their 6-month follow-up (Tables S1 and S2 in the Data Supplement).

**Table 1.**
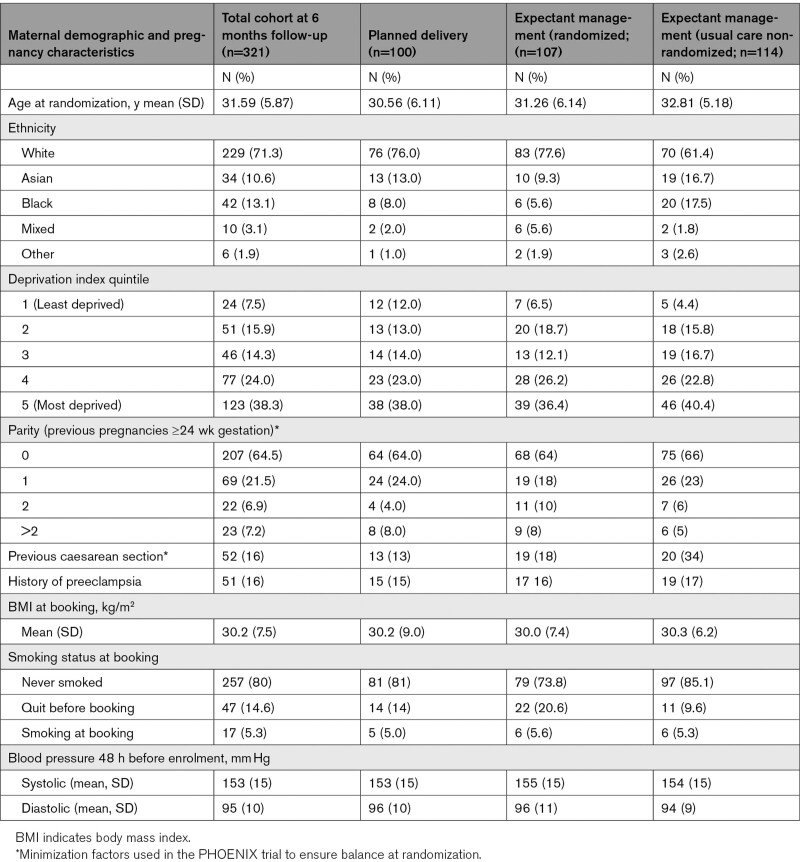
Baseline Maternal Demographic and Pregnancy Characteristics at Trial Entry on All Women Who Had 6 Month Echocardiography (n=321)

**Table 2.**
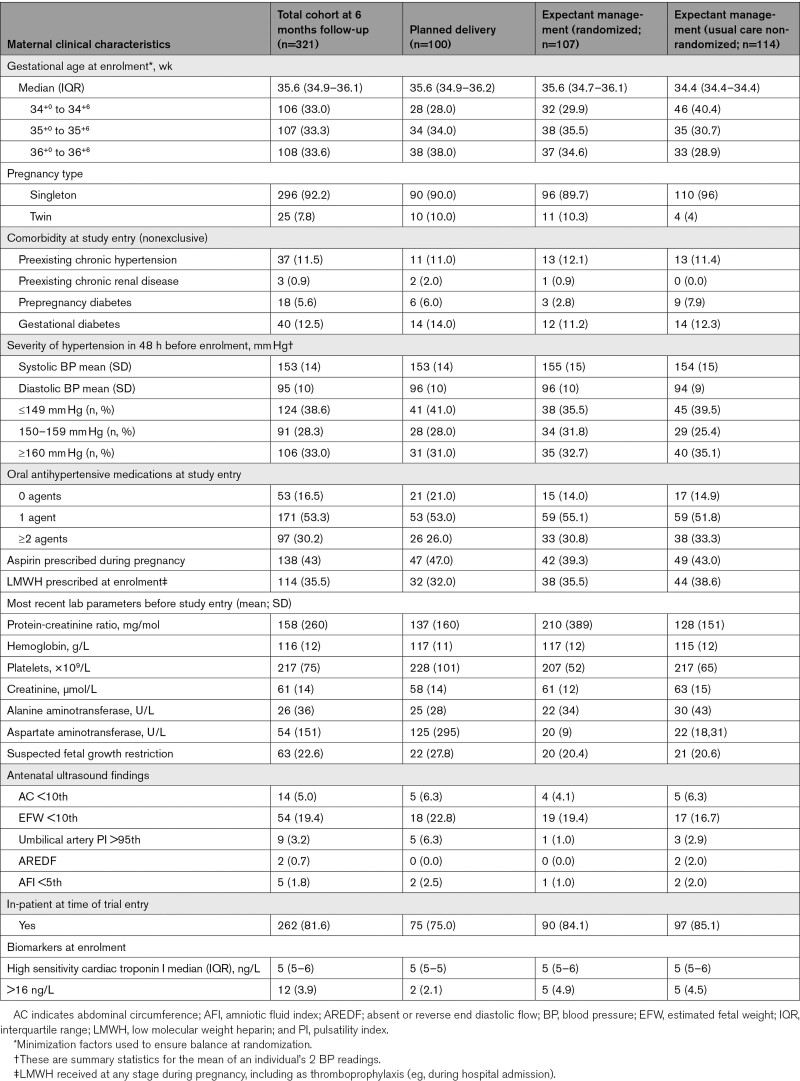
Maternal Clinical Characteristics for Women With Primary Outcome

**Figure.**
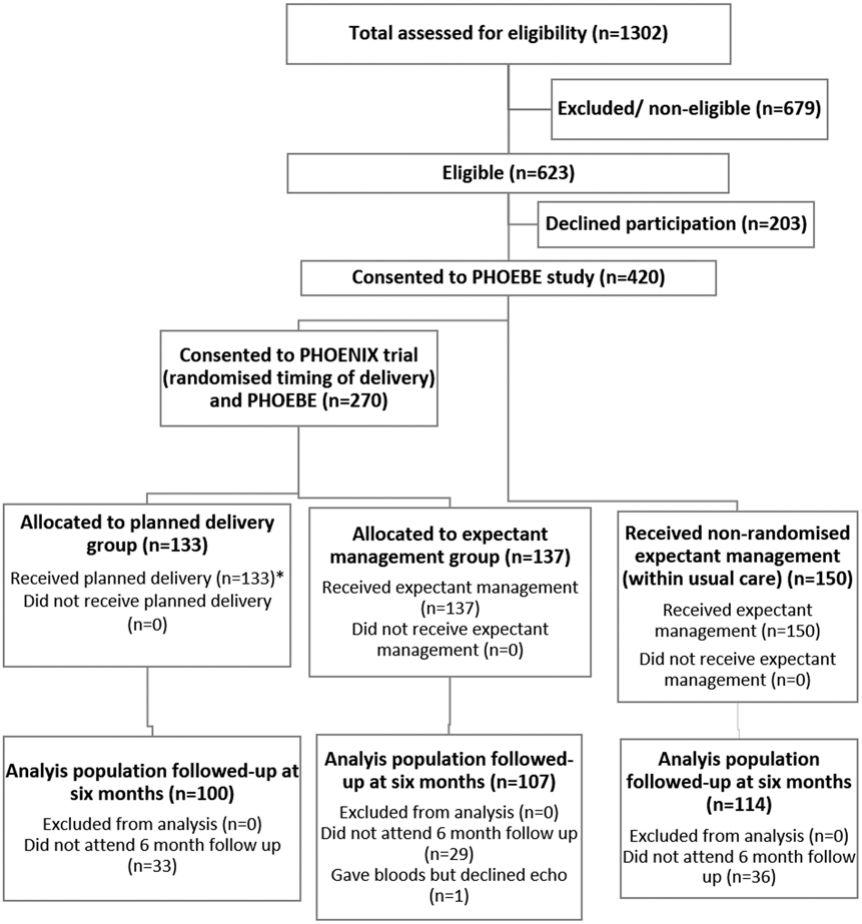
Participant flow chart.

### Outcome Data

Of the 321 women within the cohort, 51% (n=164) and 10% (n=31) had the primary outcome using 2009 and 2016 definitions, respectively. Overall, 10% (31/321) of women had an LVEF <55% 6 months postpartum. Using 2009 diastolic dysfunction sub-classification, 9% (n=27) of women had grade I, 40% (n=123) grade II and 1% (n=3) grade III dysfunction. Using the newer 2016 diastolic sub-classification, 18% (n=58), 2% (n=5), and 0% (n=1) had 1, 2, and 3 diastolic dysfunction criteria present, respectively (Table 3). Hypertension prevalence, defined as on antihypertensive treatment or systolic BP >140 mm Hg ±diastolic BP >90 mm Hg 6 months postpartum was present in 71% (n=229) of the cohort. Seventeen percent (n=50) of women had evidence of concentric remodeling and 1% (n=3) had evidence of eccentric remodeling. At 6 months postpartum, 1% (n=4) of women had a high sensitivity cTnI >16 ng/L, 13.2% (n=38) NT-proBNP >100 ng/L, and 0.7 (n=2) had a MyC >87ng/L.

**Table 3.**
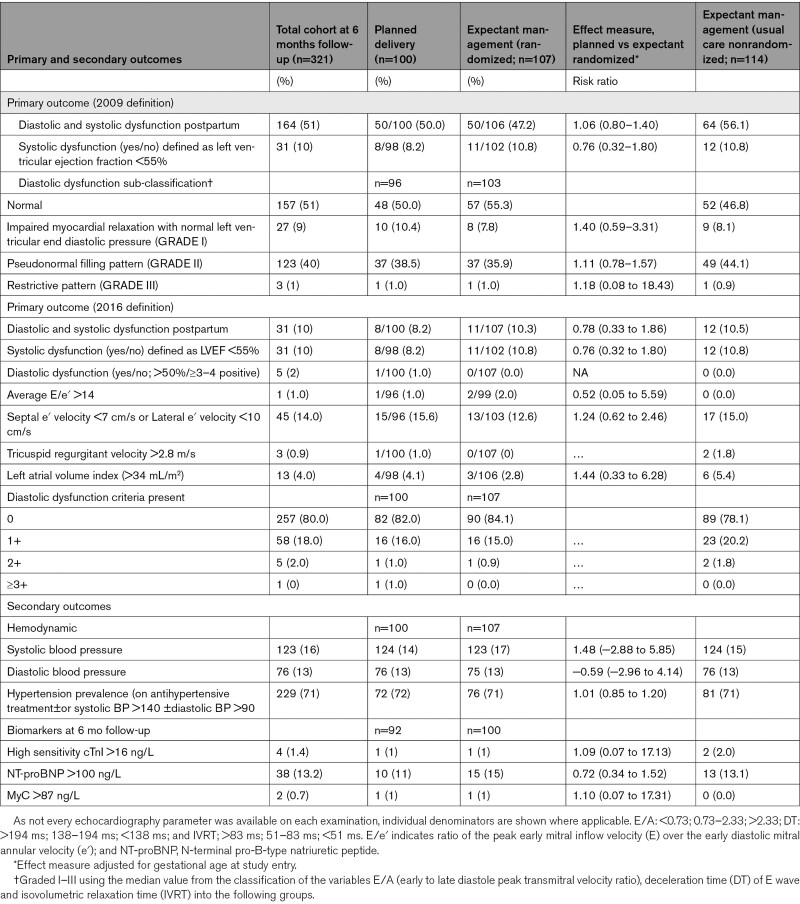
Primary and Secondary Outcomes at 6 Months Postpartum

### Nested Randomized Comparison

There were no differences between women in the planned delivery group compared with the expectant management group in the primary outcome using either the 2009 (RR, 1.06 [0.80–1.40]) or 2016 definitions (RR, 0.78 [0.33–1.86]) shown in Table 3. No between group differences were observed in 2009 diastolic dysfunction grade 1 (RR, 1.40 [0.59–3.31]), grade 2 (1.11 [0.78–1.57]), or grade 3 (1.18 [0.08–18.43]) diastolic dysfunction sub-classification nor in 2016 diastolic dysfunction classification. Using the more recent 2016 classification for systolic and diastolic dysfunction, similarly no differences were observed in systolic (RR, 0.76 [0.32–1.80]) or any of the diastolic dysfunction parameters. Hypertension prevalence 6 months postpartum was similar between those managed with planned delivery compared with those expectantly managed (RR, 1.01 [0.85–1.20]). No significant differences were observed in any of the cardiac parameters including geometric and hemodynamic parameters, LV global cardiac parameters, myocardial mechanics, and LV basal or apical parameters between those women with planned delivery compared with those expectantly managed (Table 3 and Table S3). The high prevalence of systolic and diastolic dysfunction or persistent hypertension was not explained by preexisting chronic hypertension as when these women were excluded (n=37, 11%), systolic and diastolic dysfunction was evident in 49.5% and hypertension in 68.7% of women.

Mean time from enrolment to delivery was 2.5 (SD 1.9) days in the planned delivery group compared with 6.8 (5.3) days in the expectant management group. No differences were observed between groups in cardiorespiratory outcomes before discharge from hospital nor in any systolic or diastolic BP measurements (Table 4).

**Table 4.**
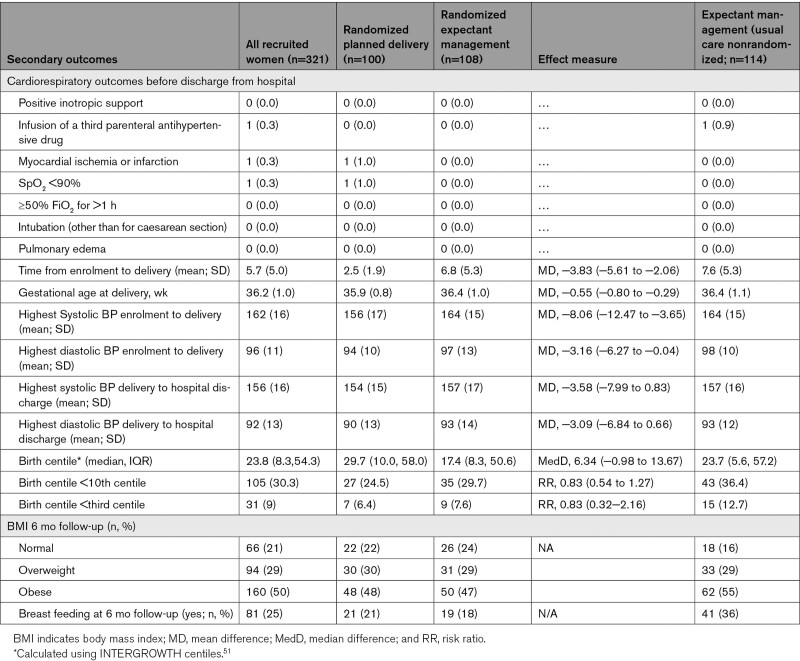
Secondary Outcomes at Discharge Following Delivery and 6 Month Follow-Up

Within the entire cohort (n=321), the only variables affecting development of systolic and diastolic dysfunction 6 months postpartum (2009 definition) was maternal body mass index (adjusted odds ratio, 1.33 [1.12–1.59] per 5 kg/m^2^) and maternal age (2.16 [1.44–3.22] per 10 years; Table 5). Interval from study enrolment to delivery was not associated with development of the primary outcome. There were no significant predictor variables for systolic and diastolic dysfunction 6 months postpartum by the updated 2016 definition (Table 5). Inclusion of antenatal high sensitivity cTnI did not alter the results.

**Table 5.**
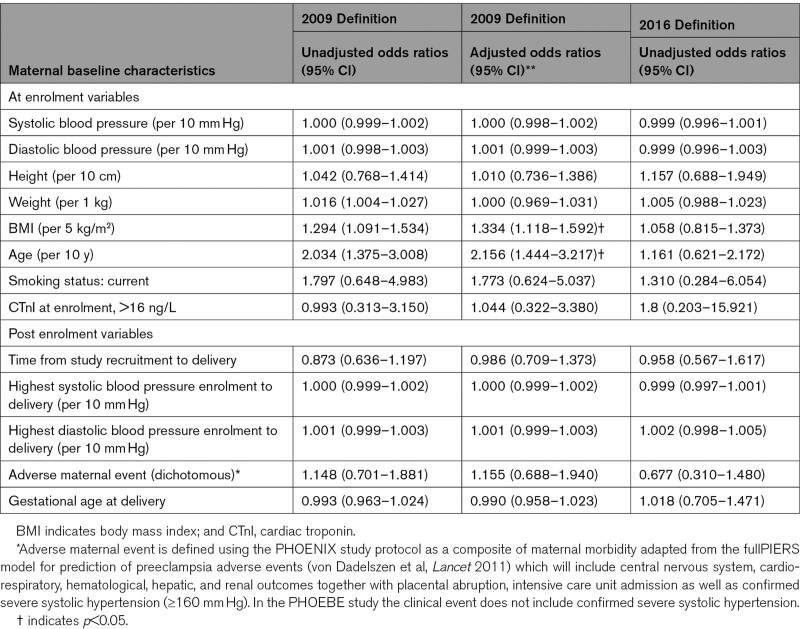
Effect of Baseline Characteristics on the Development of Cardiovascular Dysfunction (2009 and 2016 Definition) at 6 Months Postpartum (All 321 Women)

Overall, 8% (n=25) women had their clinical echocardiograms escalated by the trial cardiologist with clinical follow-up recommended. These were for a combination of structural (n=8), valvular (n=8), functional (n=9), or combined (n=2) findings. These clinical escalations accounted for 12% of those with the primary outcome (2009 definition), with 88% of those with systolic and diastolic dysfunction not requiring clinically escalation.

## Discussion

This large multicenter study highlights the substantial adverse cardiovascular sequelae of preterm preeclampsia; 10% of women with preterm preeclampsia had a LVEF <55%, 71% remained hypertensive, and 49% of women had evidence of impaired diastolic dysfunction of undetermined long-term clinical importance 6 months postpartum. In the nested randomized controlled trial of women with late preterm preeclampsia, planned delivery did not reduce cardiovascular dysfunction 6 months postpartum. Women in the planned delivery group had a mean shortening of pregnancy from enrolment to delivery of 4 days, but this did not result in decreased hypertension or cardiovascular dysfunction compared with those managed with usual care by expectant management. Only elevated body mass index and higher age at enrolment predicted the occurrence of postpartum systolic and diastolic dysfunction. The short time to delivery in the expectant arm may not have resulted in sufficient time for any longer term cardiovascular insult to be exacerbated, explaining why earlier delivery does not benefit the mother in this outcome.

This large, multicenter trial represents UK contemporaneous management of women with late preterm preeclampsia, subsequently followed up by detailed standardized BP and echocardiography assessment 6 months postpartum. Our cohort sample size (321 women) is considerably larger than any other postpartum cardiovascular study previously performed. We are unaware of any published randomized controlled trials evaluating the impact of timing of delivery on subsequent maternal cardiovascular function. The randomized design allowed us a unique opportunity to explore the impact of earlier delivery on postpartum cardiovascular function.

Strengths of this study include a sufficiently large sample of women with late preterm preeclampsia from 28 centers throughout the UK completing a detailed 6-month postpartum cardiovascular assessment to describe the burden of cardiovascular disease in this population, linking preeclampsia with longer term cardiovascular disease. The trial was conducted to rigorous standards, with a prespecified protocol without changes. Findings are likely to be generalizable to similar health care settings, because it was undertaken in a large number of maternity units across England and Wales, with diverse representation of women in terms of both demography and disease spectrum. More than half of eligible women approached agreed to participate in the trial, indicating agreement of equipoise in this scenario. Echocardiograms were performed by multiple echocardiographers throughout the UK and Wales, representative of cardiology units throughout the National Health Service. We have reported all prespecified secondary outcomes, interpreting them cautiously.

Limitations of the trial include a change in the international definition regarding systolic and diastolic dysfunction, such that interpretation of the findings need to be undertaken in the light of the prespecified 2009 definition^11^ and the later 2016 definition.^12^ Our results reflect systolic and diastolic definitions used first in 2009 and then updated in 2016. We acknowledge that there is an interim group with a LVEF between 50% and 55% and further prospective follow-up would help our understanding of the implications of this impairment in women following pregnancy. We prespecified use of the independent definition of systolic and diastolic dysfunction for this particular patient group as defined by Melchiorre et al, adapted from recommendations of the American Society of Echocardiography/European Association of Cardiovascular Imaging^11^ with adjustments for age, the higher circulating volume in pregnancy and the acute nature of preeclampsia in an otherwise previously normal cardiovascular system.^23^ Newer nonpregnant specific definitions (American Society of Echocardiography/European Association of Cardiovascular Imaging guidelines 2016)^28^ result in lower prevalence of diastolic dysfunction but cardiovascular morbidity is still evident and prevalent. There was a relatively short difference of a median 4 days in those managed with planned delivery compared with women managed expectantly, and it is likely that this difference may not have been sufficiently long to result in detectable differences in cardiovascular function 6 months postpartum. Approximately, one-third of the women recruited for this study had been eligible for, but declined participation in the main PHOENIX trial, and as a result were included as a nonrandomized expectant management (usual care) group. Results across all groups were very similar. We did not recruit women with a healthy pregnancy, as our primary research question was whether shortening of pregnancy after diagnosis of preterm preeclampsia altered the prevalence of cardiovascular dysfunction 6 months postpartum.

We considered sources of possible bias for our trial. Selection bias into the trial was unlikely due to the randomization process, which included robust allocation sequence concealment such that determining next allocation was not possible. Performance and detection bias were possible because it was not possible to mask participating clinicians or women, nor data collectors because timing of delivery was contained within maternity records where morbidity was recorded. However, the trial echocardiographer was masked to randomization groups and each echocardiogram was, therefore, read independent of knowledge of trial allocation. There was expected attrition to 6 month follow-up of around 20% in both groups, but data completeness of pregnancy outcomes was high (over 99%). The study was originally powered for an analysis of 322 women comprised of 161 women in 2 treatment groups. However, it became apparent that a group of eligible women chose not to consent to the main randomized comparison in the PHOENIX trial but would consent for the observational PHOEBE (also known as PHOENIX-3) study, with all women in this group following usual care, which was expectant management. The aims of the PHOEBE study were primarily to explore the mechanism behind the effect of the intervention (earlier timing of delivery) and to provide an understanding of postpartum cardiovascular dysfunction after preterm preeclampsia. We acknowledge that we were underpowered for examining the effect of the intervention. The primary outcome event rate was also lower than expected by both 2009 and 2016 guidelines. For the evaluation of the effect of the intervention, the 2 randomized groups were compared (as described in the Statistical Analysis Plan). As there was no signal of a significant effect in the secondary outcomes, it would suggest that we had not missed an important difference in the primary outcome (likely to be related to the much shorter separation in time between randomization and initiation of delivery between the 2 groups than anticipated). In light of this, we also combined all women recruited to provide an overall cohort of 321 women in which to complete the prognostic assessment (presented in Table 5) and to present a detailed cardiovascular assessment on a large prospective cohort of women with preterm preeclampsia. We chose 6 months as a generally accepted timepoint that pregnancy changes have resolved and underlying cardiovascular changes could not be attributed to pregnancy alone. This figure was also based on others’ work showing that persistent cardiovascular changes are present as early as 6 months postpartum.^23^ Maternal and neonatal outcomes before discharge from hospital between those randomized to immediate or expectant management are presented in Table S4 and do not differ significantly. In our original cohort, 12% had preexisting chronic hypertension. At the 6 month follow-up, 71% of women were classified as having chronic hypertension not explained by other factors other than exposure to preterm preeclampsia. 241 (75%) of women were not breast feeding and 81 (25%) of women continued to breast-feed (including as part of mixed feeding) at 6 months postpartum. Exploratory analysis demonstrated that our primary outcome was unaffected by breast-feeding status (odds ratio, 1.20 [0.78–1.87]; *P*=0.43). At the 6 month follow-up, 79% of women were classified as overweight or obese, but hypertension was seen across all weight groups. The occurrence of chronic hypertension at 6 months was not dependent on the body mass index category. The occurrence of chronic hypertension at 6 months was not dependent on the body mass index category. The proportion of women with chronic hypertension 6 months postpartum was similar across the 3 main weight categories as follows; normal weight 71% (47/66); overweight 60% (57/94); and obese 57% (91/160).

A recent systematic review summarized 36 studies of maternal cardiovascular function involving 815 women at time of disease with preeclampsia, demonstrated that increased vascular resistance and LV mass were the most consistent findings in preeclampsia.^33^ Differentiating features of a pregnancy complicated by preeclampsia from normal pregnancy include LV wall thickness of ≥1.0 cm, exaggerated reduction in early diastole/atrial contraction, and lateral e′ of <14 cm/s, markers of diastolic dysfunction. Reduced stroke volume, diastolic dysfunction, and LV remodeling are most marked in severe and early-onset preeclampsia.^23,31,34^ Our finding of cardiovascular dysfunction and persistent hypertension in the majority of women following preterm preeclampsia is in keeping with other single-center observational studies.^35–44^ However, none were multicenter nor designed to examine different maternal delivery strategies. Our finding of 71% of women with preterm preeclampsia remaining hypertensive 6 months postpartum is higher than reported in larger population based cohorts, highlighting high levels of presumed undiagnosed hypertension.^45^ As the PHOENIX trial has now reported, it is unlikely that the opportunity will arise for other investigators to examine whether timing of delivery impacts cardiovascular function using such a randomized approach. Developing accurate validated prognostic tools to best identify those at highest risk of cardiovascular dysfunction remains challenging, and postpartum intervention strategies must now be explored to reduce this cardiovascular burden of disease.

We have demonstrated that the burden of postpartum cardiovascular dysfunction following preterm preeclampsia in these women is high. In low resource health care settings where under-detected comorbidities including chronic hypertension are high and cases of fulminant eclampsia prevalent (incidence 1.4%),^46^ the burden of cardiovascular morbidity is likely much higher. Recent US data suggest stagnation in the improvements in incidence and mortality of cardiovascular disease, specifically among younger women.^47^ It is imperative that we understand the mechanisms that contribute to worsening risk factor profiles in young women to reduce future cardiovascular morbidity and mortality.

Two decades of research have documented an association between preeclampsia and major cardiovascular disorders in later life.^2,3,48,49^ Despite this body of evidence, usual practice after a pregnancy complicated by preterm preeclampsia is for no specific follow-up. It is recognized by the Joint British Societies that include the British Cardiac Society, Heart UK, and the British Hypertension Society that pregnancy and infancy are good opportunities for education and intervention.^50^ Furthermore, they endorse intensive risk factor lowering in individuals with high-risk factors that cause cardiovascular disease. Women with preterm preeclampsia are at increased risk for cardiovascular disease later in life. This study has provided mechanistic information on how subsequent clinical cardiovascular events may be mediated through impaired cardiac function identifiable at 6 months after preterm preeclampsia and highlights the postpartum period as an opportunity for early intervention before sustained and irreversible damage. There is increasing interest in the role of lifestyle interventions and therapeutic (eg, with angiotensin-converting enzyme inhibitors) to reduce subsequent cardiovascular risk. This study provides a body of evidence for postpartum cardiac functional impairment and demonstrates the need for further research into early intervention, particularly relating to novel therapeutic pathways.

## Perspectives

In conclusion, our study confirms that late preterm preeclampsia is associated with substantial postpartum cardiovascular dysfunction. The relatively short delay in those expectantly managed (compared with planned delivery) does not worsen this cardiovascular dysfunction. Further follow-up would be useful to understand the longer term cardiovascular implications of these findings and whether these parameters relate to hypertension, age, or increased body mass index. Preeclampsia should not be considered a self-limiting disease of pregnancy alone. This research improves our understanding of the mechanistic processes linking preeclampsia with maternal cardiovascular impairment. Earlier delivery does not improve or worsen postpartum cardiovascular dysfunction, and women can be reassured that prolongation of a pregnancy affected by preterm preeclampsia will not further worsen their cardiovascular health. The study informs counseling of women with preeclampsia around future risks and also identifies the postpartum period now as a critical area to target in future intervention studies.

## Acknowledgments

All authors have made substantial contributions to the research as follows: study design: F.M. McCarthy, L.C. Chappell, P. Leeson, B. Thilaganathan, P. Seed, A. Shennan, L. Poston, J. O’Driscoll. L.C. Chappell study conduct, analyses: all authors; first draft of the article: F. McCarthy; article revision and approval: all authors. We thank Ursula Bowler and Pauline Rushby (University of Oxford); Eleanor Hendy, Emma Green, Anna Brockbank, Alice Cox, (King’s College London); Linda Arnold (University of Oxford); Marcus Green (Action on Preeclampsia), and all the participating women, site research midwives and doctors for their contribution to the trial.

## Sources of Funding

The trial was funded by the National Institute for Health Research (NIHR) Efficacy and Mechanism Evaluation programme. L.C. Chappell is funded by the NIHR Professorship, RP-2014-05-019.

## Disclosures

The authors’ institutions received funding from the National Institute for Health Research (NIHR) for this work (EME 15/23/02). M. Marber is named as an inventor on a patent (WO 2010/130985 A1) held by King’s College London for the detection of cMyC as a biomarker of myocardial injury. The other authors report no conflicts.

## Supplementary Material



## Novelty and Significance

### What Is New?

- In women with late preterm preeclampsia there is persistent hypertension in 70% and systolic dysfunction in 10% at 6 months postpartum.
- The relatively short delay in those managed expectantly (compared with planned delivery) does not worsen cardiovascular dysfunction at 6 months postpartum.

### What Is Relevant?

- Routine delivery does not improve cardiovascular function, and when to deliver should depend on immediate maternal and fetal concerns.

### Summary

Preeclampsia should not be considered a self-limiting disease of pregnancy alone and optimal screening and interventional strategies should be explored and evaluated.

## References

[1] Townsend N Williams J Bhatnagar P Wickramasinghe K Rayner M. Cardiovascular Disease Statistics, 2014. 2014. British Heart Foundation 10.1136/heartjnl-2015-307516 PMC4515998 26041770

[2] Leon LJ McCarthy FP Direk K Gonzalez-Izquierdo A Prieto-Merino D Casas JP Chappell L. Preeclampsia and cardiovascular disease in a large UK pregnancy cohort of linked electronic health records: a CALIBER study. Circulation. 2019;140:1050–1060. doi: 10.1161/CIRCULATIONAHA.118.038080 31545680 10.1161/CIRCULATIONAHA.118.038080

[3] Bellamy L Casas JP Hingorani AD Williams DJ. Pre-eclampsia and risk of cardiovascular disease and cancer in later life: systematic review and meta-analysis. BMJ. 2007;335:974. doi: 10.1136/bmj.39335.385301.BE 17975258 10.1136/bmj.39335.385301.BE PMC2072042

[4] Mosca L Benjamin EJ Berra K Bezanson JL Dolor RJ Lloyd-Jones DM Newby LK Piña IL Roger VL Shaw LJ; American Heart Association. Effectiveness-based guidelines for the prevention of cardiovascular disease in women–2011 update: a guideline from the American Heart Association. J Am Coll Cardiol. 2011;57:1404–1423. doi: 10.1016/j.jacc.2011.02.005 21388771 10.1016/j.jacc.2011.02.005 PMC3124072

[5] British Heart Foundation. Coronary heart disease statistics in England, 2012. http://www.bhf.org.uk/publications/view-publication.aspx?ps=1001546

[6] Groenhof TKJ Zoet GA Franx A Gansevoort RT Bots ML Groen H Lely AT; PREVEND Group. Trajectory of cardiovascular risk factors after hypertensive disorders of pregnancy. Hypertension. 2019;73:171–178. doi: 10.1161/HYPERTENSIONAHA.118.11726 30571544 10.1161/HYPERTENSIONAHA.118.11726

[7] Zoet GA Benschop L Boersma E Budde RPJ Fauser BCJM van der Graaf Y de Groot CJM Maas AHEM Roeters van Lennep JE Steegers EAP; CREW Consortium. Prevalence of subclinical coronary artery disease assessed by coronary computed tomography angiography in 45- to 55-year-old women with a history of preeclampsia. Circulation. 2018;137:877–879. doi: 10.1161/CIRCULATIONAHA.117.032695 29459475 10.1161/CIRCULATIONAHA.117.032695

[8] Tranquilli AL Dekker G Magee L Roberts J Sibai BM Steyn W Zeeman GG Brown MA. The classification, diagnosis and management of the hypertensive disorders of pregnancy: a revised statement from the ISSHP. Pregnancy Hypertens. 2014;4:97–104. doi: 10.1016/j.preghy.2014.02.001 26104417 10.1016/j.preghy.2014.02.001

[9] NICE. Hypertension in Pregnancy: The Management of Hypertensive Disorders During Pregnancy. 2010. National Institute for Health and Care Excellence

[10] Chappell LC Brocklehurst P Green ME Hunter R Hardy P Juszczak E Linsell L Chiocchia V Greenland M Placzek A; PHOENIX Study Group. Planned early delivery or expectant management for late preterm pre-eclampsia (PHOENIX): a randomised controlled trial. Lancet. 2019;394:1181–1190. doi: 10.1016/S0140-6736(19)31963-4 31472930 10.1016/S0140-6736(19)31963-4 PMC6892281

[11] Nagueh SF Appleton CP Gillebert TC Marino PN Oh JK Smiseth OA Waggoner AD Flachskampf FA Pellikka PA Evangelista A. Recommendations for the evaluation of left ventricular diastolic function by echocardiography. J Am Soc Echocardiogr. 2009;22:107–133. doi: 10.1016/j.echo.2008.11.023 19187853 10.1016/j.echo.2008.11.023

[12] Nagueh SF Smiseth OA Appleton CP Byrd BF 3rd Dokainish H Edvardsen T Flachskampf FA Gillebert TC Klein AL Lancellotti P; Houston, Texas; Oslo, Norway; Phoenix, Arizona; Nashville, Tennessee; Hamilton, Ontario, Canada; Uppsala, Sweden; Ghent and Liège, Belgium; Cleveland, Ohio; Novara, Italy; Rochester, Minnesota; Bucharest, Romania; and St. Louis, Missouri. Recommendations for the evaluation of left ventricular diastolic function by echocardiography: an update from the American Society of Echocardiography and the European Association of Cardiovascular Imaging. Eur Heart J Cardiovasc Imaging. 2016;17:1321–1360. doi: 10.1093/ehjci/jew082 27422899 10.1093/ehjci/jew082

[13] von Dadelszen P Payne B Li J Ansermino JM Broughton Pipkin F Côté AM Douglas MJ Gruslin A Hutcheon JA Joseph KS; PIERS Study Group. Prediction of adverse maternal outcomes in pre-eclampsia: development and validation of the fullPIERS model. Lancet. 2011;377:219–227. doi: 10.1016/S0140-6736(10)61351-7 21185591 10.1016/S0140-6736(10)61351-7

[14] Lang RM Bierig M Devereux RB Flachskampf FA Foster E Pellikka PA Picard MH Roman MJ Seward J Shanewise J; American Society of Echocardiography’s Nomenclature and Standards Committee; Task Force on Chamber Quantification; American College of Cardiology Echocardiography Committee; American Heart Association; European Association of Echocardiography, European Society of Cardiology. Recommendations for chamber quantification. Eur J Echocardiogr. 2006;7:79–108. doi: 10.1016/j.euje.2005.12.014 16458610 10.1016/j.euje.2005.12.014

[15] Oxborough D Batterham AM Shave R Artis N Birch KM Whyte G Ainslie PN George KP. Interpretation of two-dimensional and tissue Doppler-derived strain (epsilon) and strain rate data: is there a need to normalize for individual variability in left ventricular morphology? Eur J Echocardiogr. 2009;10:677–682. doi: 10.1093/ejechocard/jep037 19359300 10.1093/ejechocard/jep037

[16] Batterham A Shave R Oxborough D Whyte G George K. Longitudinal plane colour tissue-Doppler myocardial velocities and their association with left ventricular length, volume, and mass in humans. Eur J Echocardiogr. 2008;9:542–546. doi: 10.1093/ejechocard/jen114 18490313 10.1093/ejechocard/jen114

[17] Dewey FE Rosenthal D Murphy DJ Jr Froelicher VF Ashley EA. Does size matter? Clinical applications of scaling cardiac size and function for body size. Circulation. 2008;117:2279–2287. doi: 10.1161/CIRCULATIONAHA.107.736785 18443249 10.1161/CIRCULATIONAHA.107.736785

[18] Lever HM Karam RF Currie PJ Healy BP. Hypertrophic cardiomyopathy in the elderly. Distinctions from the young based on cardiac shape. Circulation. 1989;79:580–589. doi: 10.1161/01.cir.79.3.580 2917389 10.1161/01.cir.79.3.580

[19] Nagueh SF. Echocardiographic assessment of left ventricular relaxation and cardiac filling pressures. Curr Heart Fail Rep. 2009;6:154–159. doi: 10.1007/s11897-009-0022-8 19723456 10.1007/s11897-009-0022-8

[20] Poppas A Shroff SG Korcarz CE Hibbard JU Berger DS Lindheimer MD Lang RM. Serial assessment of the cardiovascular system in normal pregnancy. Role of arterial compliance and pulsatile arterial load. Circulation. 1997;95:2407–2415. doi: 10.1161/01.cir.95.10.2407 9170404 10.1161/01.cir.95.10.2407

[21] Marciniak M Bijnens B Baltabaeva A Marciniak A Parsai C Claus P Sutherland GR. Interventricular interaction as a possible mechanism for the presence of a biphasic systolic velocity profile in normal left ventricular free walls. Heart. 2008;94:1058–1064. doi: 10.1136/hrt.2007.126938 17984214 10.1136/hrt.2007.126938

[22] Baltabaeva A Marciniak M Bijnens B Moggridge J He FJ Antonios TF MacGregor GA Sutherland GR. Regional left ventricular deformation and geometry analysis provides insights in myocardial remodelling in mild to moderate hypertension. Eur J Echocardiogr. 2008;9:501–508. doi: 10.1016/j.euje.2007.08.004 17905662 10.1016/j.euje.2007.08.004

[23] Melchiorre K Sutherland GR Baltabaeva A Liberati M Thilaganathan B. Maternal cardiac dysfunction and remodeling in women with preeclampsia at term. Hypertension. 2011;57:85–93. doi: 10.1161/HYPERTENSIONAHA.110.162321 21098311 10.1161/HYPERTENSIONAHA.110.162321

[24] Marciniak A Claus P Sutherland GR Marciniak M Karu T Baltabaeva A Merli E Bijnens B Jahangiri M. Changes in systolic left ventricular function in isolated mitral regurgitation. A strain rate imaging study. Eur Heart J. 2007;28:2627–2636. doi: 10.1093/eurheartj/ehm072 17526904 10.1093/eurheartj/ehm072

[25] Mor-Avi V Lang RM Badano LP Belohlavek M Cardim NM Derumeaux G Galderisi M Marwick T Nagueh SF Sengupta PP. Current and evolving echocardiographic techniques for the quantitative evaluation of cardiac mechanics: ASE/EAE consensus statement on methodology and indications endorsed by the Japanese Society of Echocardiography. Eur J Echocardiogr. 2011;12:167–205. doi: 10.1093/ejechocard/jer021 21385887 10.1093/ejechocard/jer021

[26] Kuznetsova T Herbots L Richart T D’hooge J Thijs L Fagard RH Herregods MC Staessen JA. Left ventricular strain and strain rate in a general population. Eur Heart J. 2008;29:2014–2023. doi: 10.1093/eurheartj/ehn280 18583396 10.1093/eurheartj/ehn280

[27] Brown MA Magee LA Kenny LC Karumanchi SA McCarthy FP Saito S Hall DR Warren CE Adoyi G Ishaku S; International Society for the Study of Hypertension in Pregnancy (ISSHP). The hypertensive disorders of pregnancy: ISSHP classification, diagnosis & management recommendations for international practice. Pregnancy Hypertens. 2018;13:291–310. doi: 10.1016/j.preghy.2018.05.004 29803330 10.1016/j.preghy.2018.05.004

[28] Nagueh SF Smiseth OA Appleton CP Byrd BF 3rd Dokainish H Edvardsen T Flachskampf FA Gillebert TC Klein AL Lancellotti P. Recommendations for the evaluation of left ventricular diastolic function by echocardiography: an update from the American Society of Echocardiography and the European Association of Cardiovascular Imaging. J Am Soc Echocardiogr. 2016;29:277–314. doi: 10.1016/j.echo.2016.01.011 27037982 10.1016/j.echo.2016.01.011

[29] Cantwell R Clutton-Brock T Cooper G Dawson A Drife J Garrod D Harper A Hulbert D Lucas S McClure J. Saving mothers’ lives: reviewing maternal deaths to make motherhood safer: 2006-2008. The eighth report of the confidential enquiries into maternal deaths in the United Kingdom. BJOG. 2011;118 suppl 1 1–203. doi: 10.1111/j.1471-0528.2010.02847.x 10.1111/j.1471-0528.2010.02847.x 21356004

[30] Shah ASV Anand A Strachan FE Ferry AV Lee KK Chapman AR Sandeman D Stables CL Adamson PD Andrews JPM; High-STEACS Investigators. High-sensitivity troponin in the evaluation of patients with suspected acute coronary syndrome: a stepped-wedge, cluster-randomised controlled trial. Lancet. 2018;392:919–928. doi: 10.1016/S0140-6736(18)31923-8 30170853 10.1016/S0140-6736(18)31923-8 PMC6137538

[31] Melchiorre K Sutherland GR Watt-Coote I Liberati M Thilaganathan B. Severe myocardial impairment and chamber dysfunction in preterm preeclampsia. Hypertens Pregnancy. 2012;31:454–471. doi: 10.3109/10641955.2012.697951 23030711 10.3109/10641955.2012.697951

[32] Melchiorre K Sutherland GR Liberati M Thilaganathan B. Preeclampsia is associated with persistent postpartum cardiovascular impairment. Hypertension. 2011;58:709–715. doi: 10.1161/HYPERTENSIONAHA.111.176537 21844489 10.1161/HYPERTENSIONAHA.111.176537

[33] Castleman JS Ganapathy R Taki F Lip GY Steeds RP Kotecha D. Echocardiographic structure and function in hypertensive disorders of pregnancy: a systematic review. Circ Cardiovasc Imaging. 2016;9:e004888. doi: 10.1161/CIRCIMAGING.116.004888 27609819 10.1161/CIRCIMAGING.116.004888

[34] Vaught AJ Kovell LC Szymanski LM Mayer SA Seifert SM Vaidya D Murphy JD Argani C O’Kelly A York S. Acute cardiac effects of severe pre-eclampsia. J Am Coll Cardiol. 2018;72:1–11. doi: 10.1016/j.jacc.2018.04.048 29957219 10.1016/j.jacc.2018.04.048 PMC8136241

[35] Melchiorre K Sharma R Thilaganathan B. Cardiovascular implications in preeclampsia: an overview. Circulation. 2014;130:703–714. doi: 10.1161/CIRCULATIONAHA.113.003664 25135127 10.1161/CIRCULATIONAHA.113.003664

[36] Bokslag A Franssen C Alma LJ Kovacevic I Kesteren FV Teunissen PW Kamp O Ganzevoort W Hordijk PL Groot CJM. Early-onset preeclampsia predisposes to preclinical diastolic left ventricular dysfunction in the fifth decade of life: an observational study. PLoS One. 2018;13:e0198908. doi: 10.1371/journal.pone.0198908 29894501 10.1371/journal.pone.0198908 PMC5997308

[37] Valensise H Lo Presti D Gagliardi G Tiralongo GM Pisani I Novelli GP Vasapollo B. Persistent maternal cardiac dysfunction after preeclampsia identifies patients at risk for recurrent preeclampsia. Hypertension. 2016;67:748–753. doi: 10.1161/HYPERTENSIONAHA.115.06674 26902488 10.1161/HYPERTENSIONAHA.115.06674

[38] Haas DM Ehrenthal DB Koch MA Catov JM Barnes SE Facco F Parker CB Mercer BM Bairey-Merz CN Silver RM; National Heart, Lung, and Blood Institute nuMoM2b Heart Health Study Network. Pregnancy as a window to future cardiovascular health: design and implementation of the nuMoM2b heart health study. Am J Epidemiol. 2016;183:519–530. doi: 10.1093/aje/kwv309 26825925 10.1093/aje/kwv309 PMC4782765

[39] Hwang JW Park SJ Oh SY Chang SA Lee SC Park SW Kim DK. The risk factors that predict chronic hypertension after delivery in women with a history of hypertensive disorders of pregnancy. Medicine (Baltimore). 2015;94:e1747. doi: 10.1097/MD.0000000000001747 26496291 10.1097/MD.0000000000001747 PMC4620832

[40] Ghossein-Doha C Spaanderman M van Kuijk SM Kroon AA Delhaas T Peeters L. Long-term risk to develop hypertension in women with former preeclampsia: a longitudinal pilot study. Reprod Sci. 2014;21:846–853. doi: 10.1177/1933719113518989 24440998 10.1177/1933719113518989 PMC4107566

[41] Garovic VD August P. Preeclampsia and the future risk of hypertension: the pregnant evidence. Curr Hypertens Rep. 2013;15:114–121. doi: 10.1007/s11906-013-0329-4 23397213 10.1007/s11906-013-0329-4 PMC3812434

[42] Estensen ME Remme EW Grindheim G Smiseth OA Segers P Henriksen T Aakhus S. Increased arterial stiffness in pre-eclamptic pregnancy at term and early and late postpartum: a combined echocardiographic and tonometric study. Am J Hypertens. 2013;26:549–556. doi: 10.1093/ajh/hps067 23467210 10.1093/ajh/hps067

[43] Levine LD Lewey J Koelper N Downes KL Arany Z Elovitz MA Sammel MD Ky B. Persistent cardiac dysfunction on echocardiography in African American women with severe preeclampsia. Pregnancy Hypertens. 2019;17:127–132. doi: 10.1016/j.preghy.2019.05.021 31487629 10.1016/j.preghy.2019.05.021 PMC6858847

[44] Breetveld NM Ghossein-Doha C van Kuijk SM van Dijk AP van der Vlugt MJ Heidema WM van Neer J van Empel V Brunner-La Rocca HP Scholten RR. Prevalence of asymptomatic heart failure in formerly pre-eclamptic women: a cohort study. Ultrasound Obstet Gynecol. 2017;49:134–142. doi: 10.1002/uog.16014 27404208 10.1002/uog.16014

[45] Behrens I Basit S Melbye M Lykke JA Wohlfahrt J Bundgaard H Thilaganathan B Boyd HA. Risk of post-pregnancy hypertension in women with a history of hypertensive disorders of pregnancy: nationwide cohort study. BMJ. 2017;358:j3078. doi: 10.1136/bmj.j3078 28701333 10.1136/bmj.j3078 PMC5506851

[46] Bilano VL Ota E Ganchimeg T Mori R Souza JP. Risk factors of pre-eclampsia/eclampsia and its adverse outcomes in low- and middle-income countries: a WHO secondary analysis. PLoS One. 2014;9:e91198. doi: 10.1371/journal.pone.0091198 24657964 10.1371/journal.pone.0091198 PMC3962376

[47] Wilmot KA O’Flaherty M Capewell S Ford ES Vaccarino V. Coronary heart disease mortality declines in the United States from 1979 through 2011: evidence for stagnation in young adults, especially women. Circulation. 2015;132:997–1002. doi: 10.1161/CIRCULATIONAHA.115.015293 26302759 10.1161/CIRCULATIONAHA.115.015293 PMC4828724

[48] Skjaerven R Wilcox AJ Klungsøyr K Irgens LM Vikse BE Vatten LJ Lie RT. Cardiovascular mortality after pre-eclampsia in one child mothers: prospective, population based cohort study. BMJ. 2012;345:e7677. doi: 10.1136/bmj.e7677 23186909 10.1136/bmj.e7677 PMC3508198

[49] Smith GC Pell JP Walsh D. Pregnancy complications and maternal risk of ischaemic heart disease: a retrospective cohort study of 129,290 births. Lancet. 2001;357:2002–2006. doi: 10.1016/S0140-6736(00)05112-6 11438131 10.1016/S0140-6736(00)05112-6

[50] Board JBS. Joint British Societies’ consensus recommendations for the prevention of cardiovascular disease (JBS3). Heart. 2014;100 suppl 2 ii1–ii67. doi: 10.1136/heartjnl-2014-305693 24667225 10.1136/heartjnl-2014-305693

[51] Villar J Papageorghiou AT Pang R Ohuma EO Cheikh Ismail L Barros FC Lambert A Carvalho M Jaffer YA Bertino E; International Fetal and Newborn Growth Consortium for the 21st Century (INTERGROWTH-21st). The likeness of fetal growth and newborn size across non-isolated populations in the INTERGROWTH-21st project: the fetal growth longitudinal study and newborn cross-sectional study. Lancet Diabetes Endocrinol. 2014;2:781–792. doi: 10.1016/S2213-8587(14)70121-4 25009082 10.1016/S2213-8587(14)70121-4

