# Supplementary material for: Multicenter Cohort Study, With a Nested Randomized Comparison, to Examine the Cardiovascular Impact of Preterm Preeclampsia
Source: Hypertension. 2021 Aug 30;78(5):1382–94. doi: 10.1161/HYPERTENSIONAHA.121.17171 (PMC8516808; doi:10.1161/HYPERTENSIONAHA.121.17171)
Supplement: Supplementary file 1 [file hyp-78-1382-s001.doc]

**SUPPLEMENTAL MATERIAL**

A multicentre cohort study, with a nested randomised comparison, to examine the cardiovascular impact of preterm preeclampsia

Fergus P McCarthy, ^1,2^, PhD,

Jamie M O’Driscoll,^3,4^, PhD,

Paul T Seed,^1^, MSc,

Anna Placzek, ^5^

Carolyn Gill, PhD ^1^

Jenie Sparkes, ^1^

Lucilla Poston,^1^ , PhD,

Mike Marber,^6^ , PhD,

Andrew H Shennan MD,^1^

Basky Thilaganathan,^7^, PhD,

Paul Leeson, ^8^, PhD,

Lucy C Chappell^1^, PhD.

1. Department of Women and Children's Health, King's College London, London, UK.

2. Department of Obstetrics and Gynaecology, The INFANT Research Centre, University College Cork, Cork University Maternity Hospital, Cork, Ireland.

3. School of Psychology and Life Science, Canterbury Christ Church University, Kent, UK.

4. Department of Cardiology, St George’s University Hospitals NHS Foundation Trust, London, UK.

5. National Perinatal Epidemiology Unit (NPEU), Nuffield Department of Population Health, University of Oxford.

6. Cardiovascular Division, King's College London British Heart Foundation Centre of Excellence, The Rayne Institute, St. Thomas' Hospital Campus, London, United Kingdom

7. Fetal Medicine Unit, St George's University Hospitals NHS Foundation Trust and Molecular & Clinical Sciences Research Institute, St George's University of London, UK.

8. Oxford Cardiovascular Clinical Research Facility, Radcliffe Department of Medicine, University of Oxford, United Kingdom.

Short title: Preterm preeclampsia and cardiovascular dysfunction.

Word count: 8890 words (including the title page, abstract, text, references, tables, and figures legends)

Abstract word count: 245

Number of figures: 1

Corresponding author: Dr Fergus McCarthy, Department of Obstetrics and Gynaecology, The INFANT Research Centre, University College Cork, Cork University Maternity Hospital, Wilton, Cork, Ireland.

Email; [Fergus.mccarthy@ucc.ie](mailto:Fergus.mccarthy@ucc.ie) Tel: +353 212920609 Fax: +353 212920609

Table S1: Maternal clinical characteristics for all women recruited to the PHOEBE study (n=420) presented by those who had a six month echo (n=321) and those who did not (n=99)

| **Maternal clinical characteristics** | **Cohort at six months follow-up with echo performed**  **(n=321)** | **Lost to follow up**  **(n=99)** |
| --- | --- | --- |
|  | **N (%)** | **N (%)** |
| **Age at randomisation (years mean (SD))** | 31.59 (5.87) | 30.63 (5.70) |
| **Ethnicity** |  |  |
| White | 229 (71.3) | 66 (66.7) |
| Asian | 34 (10.6) | 14 (14.1) |
| Black | 42 (13.1) | 14 (14.1) |
| Mixed | 10 (3.1) | 3 (3.0) |
| Other | 6 (1.9) | 2 (2.0) |
| **Deprivation Index quintile** |  |  |
| 1 (Most deprived) | 123 (38.3) | 43 (43.4) |
| 2 | 77 (24.0) | 29 (29.3) |
| 3 | 46 (14.3) | 15 (15.2) |
| 4 | 51 (15.9) | 7 (7.1) |
| 5 (Least deprived) | 24 (7.5) | 5 (5.1) |
| **Parity (previous pregnancies >=24 weeks’ gestation)*** |  |  |
| 0 | 207 (64.5) | 52 (52.5) |
| 1 | 69 (21.5) | 23 (23.2) |
| 2 | 22 (6.9) | 11 (11.1) |
| >2 | 23 (7.2) | 13 (13.1) |
| **Previous pregnancies <24 weeks’ gestation** |  |  |
| 0 | 66 (40.0) | 31 (50.0) |
| 1 | 61 (37.0) | 20 (32.3) |
| 2 | 24 (14.5) | 6 (9.7) |
| >2 | 14 (8.5) | 5 (8.1) |
| **Previous Caesarean section*** | 52 (16) | 19 (19) |
| **History of preeclampsia** | 51 (16) | 16 (16) |
| **BMI at booking (kg/m^2^)** |  |  |
| mean (SD) | 30.2 (7.5) | 30.7 (7.5) |
| **Smoking status at booking** |  |  |
| Never smoked | 257 (80) | 62 (62.6) |
| Quit before booking | 47 (14.6) | 47 (14.6) |
| Smoking at booking | 17 (5.3) | 16 (16.2) |
| **Blood pressure 48 hours prior to enrolment (mmHg)** |  |  |
| Systolic (mean, SD) | 153 (15) | 154 (14) |
| Diastolic (mean, SD) | 95 (10) | 93 (9) |

*Minimisation factors used in the PHOENIX trial to ensure balance at randomisation

Table S2: Maternal clinical characteristics for all women recruited to the PHOEBE study (n=420) presented by those who had a six month echo (n=321) and those who did not (n=99)

| **Maternal clinical characteristics** | **Cohort at six months follow-up with echo performed**  **(n=321)** | **Lost to follow up**  **(n=99)** |
| --- | --- | --- |
| **Gestational age at enrolment*(weeks)** |  |  |
| Mean (SD) | 35.4 (0.86) | 35.4 (0.87) |
| 34^+0^ to 34^+6^ | 106 (33.0) | 38 (38.4) |
| 35^+0^ to 35^+6^ | 107 (33.3) | 26 (26.3) |
| 36^+0^ to 36^+6^ | 108 (33.6) | 35 (35.4) |
| **Pregnancy type** |  |  |
| Singleton | 296 (92.2) | 95 (96) |
| Twin | 25 (7.8) | 4 (4) |
| **Comorbidity at study entry (non-exclusive)** |  |  |
| Pre-existing chronic hypertension | 37 (11.5) | 15 (15.2) |
| Pre-existing chronic renal disease | 3 (0.9) | 2 (2.0) |
| Pre-pregnancy diabetes | 18 (5.6) | 8 (8.1) |
| Gestational diabetes | 40 (12.5) | 17 (17.2) |
| **Severity of hypertension in 48 hours prior to enrolment**† **(mmHg)** |  |  |
| Systolic BP mean (SD) | 153 (14) | 154 (14) |
| Diastolic BP mean (SD) | 95 (10) | 93 (9.4) |
| <=149mmHg (n, %) | 124 (38.6) | 38 (38.4) |
| 150-159mmHg (n, %) | 91 (28.3) | 24 (24.2) |
| >=160mmHg (n, %) | 106 (33.0) | 37 (37.4) |
| **Oral antihypertensive medications at study entry** |  |  |
| 0 agents | 53 (16.5) | 11 (11.1) |
| 1 agent | 171 (53.3) | 16 (66.7) |
| >=2 agents | 97 (30.2) | 22 (22.2) |
| **Aspirin prescribed during pregnancy** | 138 (43) | 42 (42.4) |
| **LMWH prescribed at enrolment** | 114 (35.5) | 36 (36.4) |
| **Most recent lab parameters prior to study entry (mean; SD)** |  |  |
| Protein-creatinine ratio (mg/mol) | 158 (260) | 148 (226) |
| Haemoglobin (g/L) | 116 (12) | 115 (12) |
| Platelets (x10^9/L) | 217 (75) | 234 (72) |
| Creatinine (µmol/L) | 61 (14) | 61 (17) |
| Alanine aminotransferase (U/L) | 26 (36) | 27 (54) |
| Aspartate aminotransferase (U/L) | 54 (151) | 87 (132) |
| **Suspected fetal growth restriction** | 63 (22.6) | 18 (21.2) |
| **Antenatal ultrasound findings** |  |  |
| AC <10^th^ | 14 (5.0) | 4 (5) |
| EFW <10^th^ | 54 (19.4) | 16 (18.8) |
| Umbilical artery PI >95^th^ | 9 (3.2) | 2 (2.4) |
| AREDF | 2 (0.7) | 2 (2.4) |
| AFI <5^th^ | 5 (1.8) | 2 (2.4) |
| **In-patient at time of trial entry** |  |  |
| Yes | 262 (81.6) | 89 (89.9) |

* Minimisation factors used to ensure balance at randomisation

†These are summary statistics for the mean of an individual’s two BP readings. LMWH; Low Molecular Weight Heparin, AC; Abdominal circumference, EFW; Estimated fetal weight, PI; pulsatility index; AREDF; absent to reverse end diastolic flow, AFI; Amniotic Fluid Index.

Table S3: Additional echocardiography parameters at six month postpartum follow up.

| **Echocardiography parameters** | **Total cohort at six months follow-up**  **(n=321)** | **Planned**  **delivery**  **(n=100)** | **Expectant management (randomised)**  **(n=107)** | **Effect measure***  **Planned vs expectant randomised** | **Expectant management**  **(usual care non-randomised)**  **(n=114)** |
| --- | --- | --- | --- | --- | --- |
|  | **(%)** | **(%)** | **(%)** | **Risk Ratio** |  |
| **Echocardiography parameters** |  | n=100 | n=107 |  |  |
| Relative wall thickness (ratio) | 0.35 (0.06) | 0.35 (0.06) | 0.35 (0.07) | 0.00 (-0.02, 0.01) | 0.36 (0.07) |
| Left ventricular mass index (g/m2) | 65.1 (14.8) | 63.3 (16.4) | 66.7 (14.7) | -3.41 (-7.69, 0.87) | 65.3 (13.4) |
| LV mass (g) | 125 (32) | 122 (31) | 128 (36) | -5.73 (-14.89, 3.42) | 125 (29) |
| Stroke volume (ml) | 62.6 (11.4) | 64.1 (10.5) | 62.4 (11.6) | 1.69 (-1.37, 4.74) | 61.6 (12.0) |
| Cardiac output (L/min) | 4.7 (0.9) | 4.8 (0.9) | 4.7 (1.0) | 0.12 (-0.14, 0.39) | 4.6 (0.9) |
| **Geometric and haemodynamic parameters** |  |  |  |  |  |
| *Left ventricular geometry* |  |  |  |  |  |
| Normal | 265 (83) | 84 (84) | 88 (82) | Ref | 93 (82) |
| Concentric remodelling | 53 (17) | 14 (14) | 18 (17) | 0.84 (0.44, 1.60) | 21 (18) |
| Eccentric remodelling | 3 (1) | 2 (2) | 1 (1) | 2.07 (0.19, 22.41) | 0 (0) |
| ***LV global cardiac parameters*** |  |  |  |  |  |
| LVEF (%) | 58.5 (4.4) | 57.9 (4.7) | 58.4 (3.9) | -0.49 (-1.70, 0.70) | 59.0 (4.6) |
| E/A ratio | 1.37 (0.39) | 1.37 (0.41) | 1.40 (0.43) | -0.02 (-0.14, 0.09) | 1.35 (0.32) |
| Average E/e' | 7.07 (1.98) | 6.95 (2.07) | 7.24 (2.21) | -0.28 (-0.89, 0.32) | 7.03 (1.66) |
| Lateral e' velocity cm/sec | 0.14 (0.03) | 0.14 (0.04) | 0.14 (0.03) | 0.00 (-0.01, 0.01) | 0.13 (0.03) |
| Septal e' velocity cm/s | 0.10 (0.02) | 0.10 (0.02) | 0.10 (0.02) | 0.00 (-0.01, 0.01) | 0.10 (0.02) |
| Tricuspid regurgitant velocity (m/s) | 1.49 (0.79) | 1.46 (0.79) | 1.47 (0.83) | -0.01 (-0.23, 0.21) | 1.55 (0.75) |
| Left atrial volume index (ml/m2) | 21.1 (6.7) | 20.3 (6.6) | 21.7 (6.5) | -1.42 (-3.22, 0.39) | 21.2 (7.1) |
| **Myocardial mechanics** |  |  |  |  |  |
| *LV longitudinal parameters* |  |  |  |  |  |
| Peak global LV longitudinal strain (%) | -17.0 (3.3) | -16.9 (3.4) | -16.9 (3.2) | -0.02 (-0.95, 0.90) | -17.1 (3.4) |
| Peak global LV longitudinal strain rate(%·s^-1^) | -0.89 (0.20) | -0.89 (0.2) | -0.9 (0.2) | 0.03 (-0.03, 0.08) | -0.91 (0.23) |
| ***LV basal parameters*** |  |  |  |  |  |
| Basal radial strain (%) | 24.7 (16.0) | 24.5 (15.1) | 26.4 (18.3) | -1.95 (-6.59, 2.70) | 23.4(14.2) |
| Basal radial strain rate (%·s^-1^) | 1.40 (0.95) | 1.42 (1.13) | 1.43 (0.92) | 0.00 (-0.29, 0.28) | 1.35 (0.80) |
| Basal circumferential strain (%) | -17.57 (5.98) | -17.84 (5.82) | -17.32 (5.75) | -0.51 (-2.12, 1.09) | -17.6 (6.35) |
| Basal circumferential strain rate (%·s^-1^) | -1.10 (0.38) | -1.12 (0.37) | -1.09 (0.37) | -0.03 (-0.13, 0.07) | -1.10 (0.39) |
| ***LV apical parameters*** |  |  |  |  |  |
| Apical radial strain (%) | 24.8 (17.7) | 25.1 (13.3) | 24.9 (15.7) | -0.20 (-4.51, 4.12) | 24.4 (22.2) |
| Apical radial strain rate (%·s^-1^) | 1.3 (0.9) | 1.3 (1.1) | 1.2 (0.6) | -0.14 (-0.40, 0.12) | 1.3 (0.8) |
| Apical circumferential strain (%) | -21.6 (7.1) | -22.0 (7.3) | -21.6 (7.1) | 0.34 (-1.78, 2.47) | -21.3 (7.0) |
| Apical circumferential strain rate (%·s^-1^) | -1.3 (0.5) | -1.3 (0.5) | -1.3 (0.4) | 0.00 (-0.14, 0.14) | -1.3 (0.5) |

*Effect measure adjusted for gestational age at study entry

E/A: <0.73; 0.73-2.33; >2.33

DT: >194ms; 138-194ms; <138ms

IVRT; >83ms; 51-83ms; <51ms

E/e'; ratio of the peak early mitral inflow velocity (E) over the early diastolic mitral annular velocity (e′)

Table S4: Maternal and neonatal outcomes prior to discharge from hospital

| **Maternal and neonatal outcomes prior to discharge from hospital (n, %)** | **Randomised Planned**  **delivery**  **(n=100)** | **Randomised Expectant management**  **(n=108)** | **Effect measure (Risk ratio; 95% CI)** |
| --- | --- | --- | --- |
| Maternal death | 0 (0) | 0 (0) | N/A |
| Eclampsia | 0 (0) | 1 (1) | N/A |
| Stroke | 0 (0) | 0 (0) | N/A |
| Pulmonary oedema | 0 (0) | 0 (0) | N/A |
| HELLP syndrome | 2 (2) | 1 (1) | 2.14 (0.2,23.2) |
| Placental abruption | 0 (0) | 2 (2) | N/A |
| Postpartum haemorrhage | 12 (12) | 14 (13) | 0.92 (0.5,1.9) |
| Severe Hypertension (≥160mmhg systolic) | 53 (53) | 80 75) | 0.71 (0.6, 0.9) |
| Thromboembolism | 0 (0) | 0 (0) | N/A |
| Maternal ICU admission | 1 (1) | 1 (1) | 1.07 (0.1,16.9) |
| Neonatal death | 0 (0) | 0 (0) | N/A |
| Hypoxic Ischaemic encephalopathy | 0 (0) | 0 (0) | N/A |
| Neonatal sepsis | 0 (0) | 0 (0) | N/A |
| Neonatal hypoglycemia | 8 (8) | 13 (12) | 0.66 (0.3, 1.5) |
| Neonatal jaundice | 3 (3) | 2 (2) | 1.60 (0.3, 9.4) |
| Gestational Age at delivery (mean, SD) | 35.9 (0.78) | 36.4 (0.9) | -0.55 (-0.8, -0.3) |
| Birthweight (grams; mean, SD) | 2460 (460) | 2499 (516) | -39 (-172, 95) |
| Admission to Neonatal unit | 42 (42) | 34 (32) | 1.32 (0.9, 1.9) |
| Admission to NICU | 3 (3) | 7 (7) | 0.46 (0.1, 1.7) |
