# Supplementary figures and images for: Multicenter Cohort Study, With a Nested Randomized Comparison, to Examine the Cardiovascular Impact of Preterm Preeclampsia
Source: Hypertension. 2021 Aug 30;78(5):1382–94. doi: 10.1161/HYPERTENSIONAHA.121.17171 (PMC8516808; doi:10.1161/HYPERTENSIONAHA.121.17171)

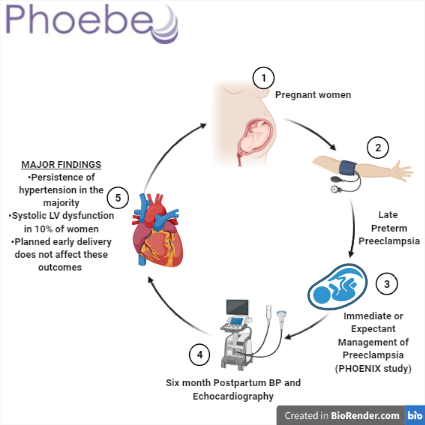

Supplement: Supplementary file 2 [file hyp-78-1382-s002.jpg]
